# Daytime napping associated with increased symptom severity in fibromyalgia syndrome

**DOI:** 10.1186/s12891-015-0464-y

**Published:** 2015-02-07

**Authors:** Alice Theadom, Mark Cropley, Thomas Kantermann

**Affiliations:** National Institute for Stroke and Applied Neuroscience, Auckland University of Technology, 90 Akoranga Drive, Private Bag 92006, Auckland, New Zealand; Department of Psychology, University of Surrey, Surrey, UK; Chronobiology Unit, Groningen institute for Evolutionary Life Sciences, University of Groningen, Groningen, The Netherlands; Institute for Occupational, Social and Environmental Medicine, Clinical Centre Ludwig-Maximilians University Munich, Ziemssenstrasse 1, 80336 Munich, Germany

**Keywords:** Daytime napping, Fibromyalgia syndrome, Sleep, Memory, Pain, Fatigue

## Abstract

**Background:**

Previous qualitative research has revealed that people with fibromyalgia use daytime napping as a coping strategy for managing symptoms against clinical advice. Yet there is no evidence to suggest whether daytime napping is beneficial or detrimental for people with fibromyalgia. The purpose of this study was to explore how people use daytime naps and to determine the links between daytime napping and symptom severity in fibromyalgia syndrome.

**Methods:**

A community based sample of 1044 adults who had been diagnosed with fibromyalgia syndrome by a clinician completed an online questionnaire. Associations between napping behavior, sleep quality and fibromyalgia symptoms were explored using Spearman correlations, with possible predictors of napping behaviour entered into a logistic regression model. Differences between participants who napped on a daily basis and those who napped less regularly, as well as nap duration were explored.

**Results:**

Daytime napping was significantly associated with increased pain, depression, anxiety, fatigue, memory difficulties and sleep problems. Sleep problems and fatigue explained the greatest amount of variance in napping behaviour, p < 0.010. Those who engaged in daytime naps for >30 minutes had higher memory difficulties (t = −3.45) and levels of depression (t = −2.50) than those who napped for shorter periods (<30mins) (p < 0.010).

**Conclusions:**

Frequent use and longer duration of daytime napping was linked with greater symptom severity in people with fibromyalgia. Given the common use of daytime napping in people with fibromyalgia evidence based guidelines on the use of daytime napping in people with chronic pain are urgently needed.

## Background

Fibromyalgia syndrome (FMS) is a chronic medical condition associated with an amplification of pain signals in the central nervous system and decreased ability of the nervous system to inhibit concomitant pain responses [1,2]. In Europe, FMS has been estimated to affect approximately 4.7% of the general population, [3] with a high prevalence in females (female: male ratio of 9–10:1) [4-6]. FMS is a disabling condition with patients experiencing high levels of widespread persistent pain, fatigue, cognitive impairment and sleep disturbance that makes it difficult to engage in everyday activities [7,8].

In a qualitative study exploring sleep disturbance in people with FMS, it was revealed that daytime napping was used as a coping strategy to manage the impact of poor sleep and replenish energy levels to enable participants to continue their daily activities [9]. Daytime napping is commonly used across cultures to recuperate and counteract the effects of sleep deprivation [10]. In healthy adults, it has been revealed that naps of <30 minutes lead to improvements in alertness, emotional state and cognitive performance [11-16]. Underlying circadian processes have been found to influence the benefits achieved following a nap, with the greatest restorative effects revealed if naps are taken on a regular basis in the afternoon when the most severe sleepiness and pressure for sleep is likely to occur [10,17]. Therefore, it appears that the duration of naps and the time of day people engage in a daytime nap is linked to its effects.

Despite the evidence of the beneficial effects of napping found in healthy adults in terms of improved alertness, emotional state and cognitive performance, the impact of napping in clinical populations has received little attention. Indeed in the pain management context, many patients are currently advised to avoid napping during the day due to concerns over disrupting nocturnal sleep patterns [18-21]. Given the interrelatedness of symptoms in FMS, it is likely that daytime napping is intrinsically linked with other symptoms of the condition [22,23]. Consequently, this study aimed to explore the frequency and type of napping used by people with FMS and to explore any associations between daytime napping and symptoms of the condition.

## Methods

### Participants

Participants were eligible to take part in the study if they were aged >18 years and had been diagnosed with FMS by a GP or consultant. Participants were asked to provide details of their FMS diagnosis for verification. Participants who were undertaking shift-work or who had been diagnosed with other specific sleep disorders (e.g., sleep apnea or restless legs syndrome) were excluded. Participants with other medical, mood or psychiatric disorders were retained in the analysis to ensure generalizability with the FMS population where comorbidities are common.

### Procedure

Participants were recruited through advertisements disseminated by the Fibromyalgia Association UK, and FibroAction UK. The advertisement directed participants to an online information sheet and consent form. Once consent was obtained, participants were asked to complete the online questionnaire. Responses on the online questionnaire were collected between September 2010 and February 2011. The study received ethical approval from the University of Surrey Departmental Ethics Committee. Participants were asked to confirm their consent to take part in the study by ticking a box after being informed of the study process.

Demographic information including age, sex, marital status, time since diagnosis, co-morbid conditions, employment status and number of children living in the household, was collected to describe the sample population and to explore the factors that may influence daytime napping and sleep quality. To assess daytime napping behaviour and FMS symptoms, a range of measures were included within the online questionnaire. To enable the exploration of the impact of regular napping behaviour on symptoms in FMS, participants were classified into two categories, those who reported regularly taking a daytime nap at least once a day (or more often), and those who engaged in naps less frequently.

### Assessments

Daytime napping behaviour was assessed by asking participants how often they took a daytime nap (> once a day, once a day, once a week, once a month, or rarely/never). Participants were also asked how long a nap would typically last (≤15 minutes, 16–30 minutes, 31–45 minutes, 46–60 minutes, >60 minutes), whether they planned their naps, their reasons for taking a nap and the time of day they typically took a nap (morning, afternoon or evening).

Nocturnal sleep quality was assessed using the Medical Outcomes Study (MOS) Sleep Scale [24]. The MOS consists of 12 items exploring the quality of nocturnal sleep. Two subscales explore any sleep problems experienced (Sleep Problems Index I and II). Subscale scores were calculated by transforming the raw scores to values between 0–100. The MOS has been demonstrated to be a well-validated and reliable measure in different clinical populations, [24-26] and was recommended for use in the FMS population by Salaffi et al. [27].

Fatigue was measured by the Fatigue Severity Scale (FSS) [28]. This scale contains nine items that ask about the severity and impact of fatigue over the preceding week. Each item is rated on a scale ranging from 1 (strongly disagree) to 7 (strongly agree). Total scores are calculated by summing the item responses to yield a score between 9–63. The FSS has previously been used in FMS, [27] and has been shown to have high internal reliability, [28] and validity [29].

Levels of subjective pain were assessed by using the McGill Short Form Pain Questionnaire [30]. Participants were presented with 15 words (items) describing different characteristics of pain and were asked to rate how much they experience each type of pain from 0 (none) to 3 (severe). Each item was categorized into one of two subscales (11 sensory items and 4 affective pain items), with the ratings for each subscale added to yield a total score between 0–33 and 0–12, respectively. An overall pain rating was calculated by summing all responses on the 15 items to yield a score between 0–45.

Mood disturbance was assessed using the Hospital Anxiety and Depression Scale (HADS) [31,32]. This scale has been widely used for assessing levels of anxiety and depression in patients with medical problems. The scale consists of 14 questions that ask participants how often they have experienced each item (e.g., I feel tense and wound up) over the past week on a scale of 0 (not at all) to 3 (most of the time). There are two subscales (anxiety and depression) and subscale scores range between 0–21 (0–7 normal, 8–10 mild, 11–14 moderate and 15–21 representing a severe level of anxiety/depression).

Memory was assessed using the Everyday Memory Questionnaire-Revised (EMQ-R) [33]. This measure contains 13 items which explore memory impairment in situations that occur in everyday life. Participants were asked to respond to each item by stating how often the given situation has happened in the last month on a scale of 0 (once or less per month) to 4 (once or more in a day). Total scores were calculated by summing the item scores (ranging from 0–52). To enable comparisons with previous publications, the average item score has also been reported in the current study.

### Statistical analyses

All data were analyzed using SPSS Version 19.0 (SPSS Inc., Chicago, IL, USA). Missing values were replaced with participant mean substitution. Statistical significance was considered at a two-tailed level of p < 0.01 to account for the multiple statistical tests conducted.

Descriptive statistics were used to describe the sample and distribution of scores on the assessment measures. For variables with a skewed distribution (>0.3), medians and interquartile ranges were reported, with Mann Whitney U tests used to compare median values on outcome measures between groups. For variables with data meeting parametric assumptions, mean and standard deviations were reported with t-tests used to detect any differences between groups. Chi Square tests were used to explore differences on categorical data. Group differences were explored between daily and non-regular nappers and also for examining differences in napping behaviour within the daily napping group (those who nap for less than or more than 30 minutes and also for non-intentional versus intentional nappers). Higher scores on all outcome measures indicate poorer outcome.

A logistic regression analysis was conducted to explore the impact of predictors of engaging in daily napping behaviour. Variables were entered into the regression model using the forward conditional method, to allow identification of the variables that contribute most to the model.

## Results

### Sample characteristics

**Figure 1:**
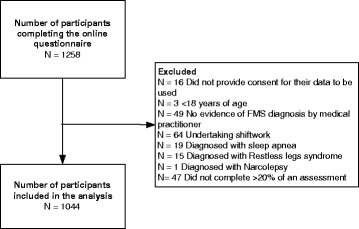
**Participants flow diagram.**

A total of 1044 participants met the inclusion criteria for this study and were included in the final analysis (Figure 1). There was a high percentage of females in this sample (92.5%), reflecting the increased prevalence of FMS in the female population. Participants were aged between 18 and 88 years, with the time in years since diagnosis ranging between 0 and 30 years. Four percent of the sample were excluded from the analysis because they completed less than 80% of items on one or more of the measures. The excluded 4%, were not found to be significantly different from the participants who did complete the full questionnaire on age, gender and time since diagnosis.

There was a high rate of co-morbid conditions (Table 1), with the most frequent conditions including arthritis (20%), irritable bowel syndrome (15%) and asthma (13%). Of the sample, 5.1% reported a psychiatric condition and 11.2% had depression. Due to the wide range of co-morbidities reported, participants were classified as having no co-morbidity (N = 203), 2 or less co-morbidities (N = 548) or >2 co-morbidities (N = 293). In addition, a large proportion of the sample were taking medication to relieve their FMS symptoms, including painkillers (opiods), non-steroidal anti-inflammatory medications and tricyclic antidepressants.

**Table 1:** **Sample characteristics**

	**Median**	**Interquartile range**
Duration since symptom onset in years	7.0	10.0
Years since diagnosis	3.0	6.0
**McGill Pain questionnaire** (range 0–45)	30.0	11.0
	**Frequency**	**Percentage**
**Marital status**		
Married or living with partner	733	70.2
Separated/divorced/widowed	192	18.4
Single	119	11.4
**Taking medication for FMS**	885	84.8
**Diagnosed co-morbid condition/s**	844	80.8
**In current employment**	386	37.0
**Napping behaviour**		
Regularly nap once a day or more	408	39.1
Occasionally or never nap	636	60.9
	**Mean**	**Standard deviation**
**Age**	46.9	10.4
**MOS Sleep problems index I** (range 0–100)	65.2	17.0
**MOS Sleep problems index II** (range 0–100)	66.9	16.7
**Fatigue severity score** (range 9–63)	55.3	9.9
**HADS Anxiety subscale** (range 0–21)	12.2	4.4
**HADS Depression subscale** (range 0–21)	10.4	4.1
**EMQ-R Everyday memory**		
Total memory score (range 0–52)	32.74	13.5
Item score (Mean) (range 0–4)	2.52	1.0

Analysis of only those participants who napped on a daily basis revealed that on a typical weekday 18.9% napped in the morning, 58.8% in the afternoon and 25.0% in the evening. It was revealed that a high proportion of participants found themselves taking a nap without intending to (86.5%). There were N = 235 (22.5%) who reported that they planned when they took a daytime nap sometimes or almost always. The most common reasons for taking a nap included tiredness/exhaustion (94.1%), feeling unwell (67.2%), to catch up from the previous night’s poor sleep (59.6%), due to headache (42.6%) and pain (26.2%).

As sleeping patterns naturally change as people age, napping behaviour was explored between younger adults (N = 362) and those aged more than 60 years (N = 46). Younger adults reported taking more daytime naps each day (22.4% reporting taking more than one nap per day in comparison to 13% of older adults) and a greater proportion of people napping for longer (75,7% >30 minutes in comparison to 41.3% of adults). Frequency of intentional napping was similar for both younger and older adults (32.3% and 32.6% respectively). Reasons for napping were broadly similar, although more younger adults reported napping due to pain (62.4 and 54.3% respectively) and feeling irritable (65.4% and 52.2%), with a greater proportion of older adults using napping to help them feel revitalized (younger adults 47.5% and 65.2% of older adults).

### Relationships between daytime napping and FMS symptoms

Bivariate correlations revealed that daytime napping was significantly positively associated with the number of co-morbidities rho = .12, levels of fatigue rho = .22, and pain rho = .19, anxiety rho = .12, depression rho = .18, sleep problems rho = .26, and frequent everyday memory difficulties rho = .18, p < 0.01.

To explore if medication use may be impacting on our findings, respective associations were explored between daytime napping and type of medication used. Daytime napping was significantly positively correlated with use of serotonin-norepinephrine reuptake inhibitors or selective serotonin reuptake inhibitors, rho = .10, Pregabalin or Gabapentin, rho = .10 and opioids, rho = .14 (p < 0.01), but not with other types of medications (tricyclic antidepressants, non-steroidal anti-inflammatories, hypnotics and other medications).

### Differences between regular daily nappers and those who nap less regularly

To explore differences in symptom severity in participants who regularly engaged in daily napping behavior and those who napped less regularly or not at all, differences between scores across the assessment measures were calculated (Table 2). It was revealed that participants who regularly napped on a daily basis had a higher number of comorbidities, higher levels of pain, and fatigue, memory difficulties, sleep problems, anxiety and depression (p < 0.001).

**Table 2:** **Comparisons between those who nap daily on a regular basis and those who do not**

	**Regular daily nap (N = 408)**	**Non regular nap (N = 636)**	**Test of difference**
	**Median (Interquartile range)**		
Number of children	1.00 (2.00)	0.00 (2.00)	U = 3.02, P = 0.82
Years since diagnosis	7.00 (10.00)	7.00 (9.00)	U = 0.50, p = 0.52
**McGill Pain questionnaire**			
Sensory subscale (Median, IQR)	24.00 (7.00)	22.00 (7.00)	U = 102,640, p = 0.00
Affective subscale (Median, IQR)	8.00 (4.00)	7.00 (4.00)	U = 105,928, p = 0.00
Total score median (Median, QR)	32.00 (9.75)	29.00 (11.00)	U = 101,372, p = 0.00
	**Frequency (%)**		
Male gender	41 (10%)	37 (5.8%)	χ = 6.44, p = 0.01
**Number of co-morbidities**			
None	63 (15.4%)	140 (22.0%)	Χ = 13.83, p = 0.00
≤2 Co-morbidities	207 (50.7%)	341 (53.6%)	
>2 Co-morbidities	138 (33.8%)	155 (24.4%)	
	**Mean**		
	**(Standard deviation)**		
Age	47.17 (10.08)		46.75 (10.59)
**MOS Sleep outcomes scale**			
Sleep duration in hours	5:47 (01:57)	6:04 (01:48)	t = 2.45, p = 0.02
Sleep problems index I	70.65 (15.61)	61.65 (16.95)	t =8.63, p = 0.00
Sleep problems index II	72.20 (15.28)	63.42 (16.71)	t =8.57, p = 0.00
**FSS Fatigue severity**			
Total score	57.67 (8.42)	53.72 (10.47)	t = −6.41, p = 0.00
**EMQ-R Everyday memory**			
Total score	35.61 (12.86)	30.91 (13.51)	t = −5.59, p = 0.00
**HADS Mood**			
Anxiety subscale	12.84 (4.27)	11.77 (4.37)	t = −3.89, p = 0.00
Depression subscale	11.36 (3.90)	9.83 (14.14)	t = −5.94, p = 0.00

### Differences between those who take long or short naps daily

To elucidate if the length of time people napped also affected the impact of daytime napping, differences between those napping for <30 minutes and those >30 minutes each day were calculated. Of the participants who reported taking a daytime nap at least once per day, participants who typically napped for longer than 30 minutes were younger in age, with children living in the household and had significantly higher levels of depression and memory difficulties, in comparison to participants who napped for shorter periods of time (Table 3). It was also observed that those who did not intend to nap had higher levels of pain and depression than intentional nappers.

**Table 3:** **Differences in outcome measures between different napping behaviour for those who engage in a daytime nap at least once per day**

	**Duration of daytime nap**			**Intentional versus non-intentional napping**		
	**<30 minutes (N = 115)**	**>30 minutes (N = 203)**	**Test of difference**	**Intentional N = 132**	**Non-intentional N = 276**	**Test of difference**
Age (Mean)	50.63 (10.73)	45.82 (9.50)	t = 4.44, p = 0.00	47.67 (9.40)	46.94 (10.40)	t = 0.71, p 0.48
Sex (% Male)	8 (7.0%)	33 (11.3%)	Χ = 1.6, p = 0.19	12 (9.10)	29 (10.5)	X = 0.20, p = 0.66
Median number of children	0.00 (1.00)	1.00 (2.00)	U = 9.69, p = 0.03	1.00 (2.00)	0.00 (2.00)	U = 0.00, p = 1.00
Median years since diagnosis	7.00 (9.00)	8.00 (10.00)	U = 16,902, p = 0.84	3.5 (5.0)	3.0 (5.0)	U = 0.01, p = 0.98
**Number of co-morbidities**						
None (N, %)	17 (14.8%)	46 (15.7%)	Χ =0.34, p = 0.84	17 (12.9)	46 (16.7)	X = 1.20, p = 0.55
≤2 comorbidities (N, %)	61 (53.0%)	146 (49.8%)		71 (53.8)	136 (49.3)	
>2 comorbidities (N, %)	37 (32.2%)	101 (34.5%)		44 (33.3)	94 (34.1)	
**MOS Sleep outcomes scale**						
Mean sleep duration in hours	5:32 (01:38)	5:52 (02:03)	t = −1.61, p = 0:11	6:02 (1:58)	5:39 (1:56)	t = −1.78, p = 0.08
Mean sleep problems index I	69.94 (16.45)	70.93 (15.29)	t = −0.58, p = 0.57	50.78 (16.34)	46.87 (14.48)	t = 3.55, p = 0.00
Mean sleep problems index II	70.98 (16.54)	72.68 (14.76)	t = −1.01, p = 0.31	45.03 (12.73)	42.00 (10.84)	t = 4.02, p = 0.00
**McGill Pain questionnaire**						
Median sensory subscale	25.00 (8.00)	24.00 (6.50)	U = 16,817, p = 0.82	22.00 (11.00)	25.00 (7.00)	U = 21.11, p = 0.00
Median affective subscale	8.00 (4.00)	8.00 (4.00)	U = 17,714, p = 0.81	7.5 (4.0)	8.00 (4.00)	U = 4.35, p = 0.04
Median total score	33.00 (11.00)	32.00 (10.00)	U = 17,342, p = 0.64	30.00 (9.00)	31.98 (9.00)	U = 22.45, p = 0.00
**FSS Fatigue severity**						
Mean total score	56.72 (9.09)	58.05 (8.13)	t = −1.43, p = 0.15	57.67 (8.36)	57.67 (8.57)	t = 252.45, p = 1.00
**EMQ-R Everyday memory**						
Mean total score	32.15 (14.07)	36.97 (12.11)	t = −3.45, p = 0.00	29.55 (6.64)	31.98 (7.08)	t = 272.37, p = 0.10
**HADS Mood**						
Mean anxiety score	12.13 (4.44)	13.11 (4.18)	t = −2.10, p = 0.04	12.30 (4.05)	13.12 (4.39)	t = 2.22, p = 0.03
Mean depression score	10.59 (3.99)	11.66 (3.84)	t = −2.50, p = 0.01	10.75 (3.42)	11.60 (4.01)	t = 2.72, p = 0.01

IQR = Interquartile range SD = Standard deviation.

### Predictors of daily napping behaviour

Variables significantly correlated with napping outcome were entered into a logistic regression model using the forward conditional method. As both sleep problem indexes (Index I and II of the MOS) were highly correlated with each other, only the more comprehensive sleep problems index (Index II) was entered into the regression model. The Nagelkerke R square of 0.91 indicated a strong relationship between predictors and group membership of those who napped daily or not. The overall success rate of the model to correctly classify participants was 65.4%. The Wald criterion demonstrated that sleep problems contributed most to the variance in the model (Table 4). Age, number of children living in the household and number of comorbidities, use of Pregabalin or Gabapentin or antidepressants (yes or no), levels of anxiety, depression, pain and everyday memory did not significantly contribute to the final model. Being male, sleep problems and fatigue significantly predicted daytime napping behavior in the final model (p < 0.010).

**Table 4:** **Logistic regression analyses**

	**B**	**Standard error**	**Wald**	**Sig.**	**Exp (B)**	**Lower confidence interval (95%)**	**Upper confidence interval (95%)**
Gender	−0.72	0.25	8.25	0.00	0.49	0.30	0.80
Use of opiods	0.36	0.14	6.81	0.01	1.43	1.09	1.88
Sleep problems	0.03	0.01	36.92	0.00	1.03	1.02	1.04
Fatigue	0.04	0.01	18.14	0.00	1.04	1.02	1.06
Constant	−3.99	0.55	52.57	0.00	.02		

## Discussion

The findings of this study revealed that daytime napping was associated with greater symptom severity in people with FMS. Participants who regularly took a daytime nap were found to have a higher number of co-morbidities, increased levels of pain, fatigue, sleep problems, memory difficulties, and mood disturbance in comparison to participants with FMS who napped less regularly or not at all. The duration of naps was found to have an influence on the impact of symptoms, with participants who napped >30 minutes on a daily basis found to be younger, to have children living in the household and to have greater levels of depression and memory difficulties. Sleep problems and levels of fatigue contributed most to the prediction of engaging in daytime naps in participants with FMS.

The most common reasons for napping included tiredness/exhaustion, feeling unwell, to catch up from the previous night’s poor sleep, and in response to having a headache and high levels of pain, supporting the notion that napping is used as a coping strategy in FMS. It is of concern that a high proportion of people taking a nap regularly on a daily basis, did so without intending to, suggesting the influence of underlying sleep processes on napping behaviour and people’s difficulty in remaining wakeful during the day. This was also highlighted through the findings that those who did not intent to nap experienced higher levels of pain and depression. The high frequency of taking a nap in the afternoon suggests that this may be a particularly challenging time of the day for people with FMS, when they are fatigued after expending their energy resources during the morning, in addition to the increase in sleep pressure experienced at this time of day. This suggests that patients need support in managing afternoon periods and offers support to the proposal that there is a need to tailor the recommended timing of sleep to individual sleep chronotypes and sleep patterns [17,34].

The finding that men nap more frequently than women, and that longer duration of naps were associated with poorer health outcomes such as increased depression, supports previous studies of napping behaviour in older adults [35]. Previous studies have revealed that frequency of napping behaviour increases with age [36]. However, it was interesting that in this study, age was not found to be positively associated or predictive of daytime napping behaviour in FMS, even with 11.5% of the sample aged over 60 years. Moreover, it was observed that younger adults with FMS napped more frequently and for longer periods of time than older adults; with a higher proportion of younger adults reporting napping due to pain and irritability. These findings suggests that napping behaviour may be more intrinsically linked to FMS symptoms than other demographic factors, however causality was unable to be determined within this study. This highlights the potential unique characteristics of napping behaviour in clinical populations.

Levels of sleep difficulties, fatigue and pain were revealed to be high, consistent with other studies exploring the symptom profile of people with FMS [7,37-39]. In addition, medication was found to be linked to frequency of daytime napping and may reflect the higher levels of symptoms experienced by participants. As a high proportion of people with FMS require medication, excluding such participants from the study would have biased the sample. However, due to the wide range of medications taken to manage symptoms of the FMS, quantifying medication use proved challenging. The impact of medication use was therefore explored in this study by looking at the impact of different types of medication on napping behaviour, however more detailed investigation on the effect of varying dosages and brands of medication is warranted.

This study only explored napping behaviour within those who napped on a daily basis due to the difficulties in accurately assessing napping behaviour used on a less frequent basis which can highly variable [40]. Self-reported napping behaviour has been found to underreport actual napping behaviour, and therefore the present findings may well underestimate actual napping behaviour. Studies using daily sleep diaries or objective measurements of daytime napping such as wrist actigraphy are needed to support our findings. And more objective methods will assist in determining causality of associations identified and aid our understanding of the impact of less regular napping behavior on FMS symptoms. Although participants identified as having a sleep disorder were excluded from this study, it is recognized that a high proportion of sleep disorders are undiagnosed which may have influenced our findings. Screening tools for sleep disorders would also be useful to include in further research studies of sleep and FMS.

The findings that daytime napping, nocturnal sleep, medication use and symptoms of FMS including fatigue were highly interlinked suggests that there is a need to address the issue of daytime napping in pain management programmes and specific interventions may need to be developed. It is apparent that there is a lot of conflicting advice currently being given to patients, with 23% of participants being recommended to take a daytime nap by a health professional whilst others received no advice or were advised not to nap. It is important that clinical advice is informed by the research evidence and further research is needed to determine whether taking a daytime nap is a beneficial coping strategy or not and if it is, how to use napping most effectively (e.g. time of day and duration of nap) to inform clinical recommendations [41].

To date, the diagnosis of FMS is based on the American College of Rheumatology (ACR) criteria [6]. Recently, a number of limitations of these criteria have been published suggesting revising these diagnostic procedures to better accommodate the full range of symptoms associated with FMS. However, these revised criteria still require further investigation and validation in clinical practice [42]. In response to these challenges with diagnostic criteria, participants were recruited into the current study if they had been diagnosed by a clinician, and were required to provide details of their diagnosis for verification. This approach may therefore be subject to inconsistencies in diagnostic accuracy between clinicians. Despite these limitations, the current study is strengthened by its large sample size and inclusion of both men and women with FMS. This study clearly highlights the need to increase understanding the role of daytime napping in people with FMS and its likely impact on other clinical populations experiencing high levels of poor sleep, pain and fatigue.

## Conclusions

Greater frequency and duration of daytime napping was associated with more severe symptomology in FMS. The majority of participants reported using daytime napping as a strategy for coping with poor sleep and FMS symptoms. Further research is needed to understand if daytime napping is detrimental to symptom severity or if it can be recommended as a strategy to manage symptoms in FMS.

## Abbreviations

FMS: Fibromyalgia syndrome
MOS: Medical outcomes study sleep scale
FSS: Fatigue severity scale
McGill: McGill pain questionnaire
HADS: Hospital anxiety and depression scale
EMQ-R: Everyday memory questionnaire revised
IQR: Interquartile range
SD: Standard deviation
CI: Confidence interval
